# Draft Genome Sequences of *Elizabethkingia anophelis* Strains R26^T^ and Ag1 from the Midgut of the Malaria Mosquito *Anopheles gambiae*

**DOI:** 10.1128/genomeA.01030-13

**Published:** 2013-12-05

**Authors:** Phanidhar Kukutla, Bo G. Lindberg, Dong Pei, Melanie Rayl, Wanqin Yu, Matthew Steritz, Ingrid Faye, Jiannong Xu

**Affiliations:** Biology Department, New Mexico State University, Las Cruces, New Mexico, USAa; Department of Molecular Biosciences, the Wenner-Gren Institute, Stockholm University, Stockholm, Swedenb

## Abstract

*Elizabethkingia anophelis* is a species in the family *Flavobacteriaceae*. It is a dominant resident in the mosquito gut and also a human pathogen. We present the draft genome sequences of two strains of *E. anophelis*, R26^T^ and Ag1, which were isolated from the midgut of the malaria mosquito *Anopheles gambiae*.

## GENOME ANNOUNCEMENT

The mosquito gut accommodates a diverse microbiota (1–4). *Elizabethkingia* sp. has been identified as a dominant resident in the gut of *Anopheles gambiae* (1, 5, 6) and *Anopheles stephensi* (3). Recently, Kämpfer and collaborators described the *Elizabethkingia anophelis* type strain R26, isolated from the midgut of the mosquito *Anopheles gambiae*, as a novel taxon in the genus *Elizabethkingia* (7). Another strain, designated Ag1, was isolated from the midgut of the mosquito *Anopheles gambiae* G3 strain in the Xu laboratory at New Mexico State University, and the strain was identified as *Elizabethkingia anophelis* based on the bacterial 16S rRNA gene sequence (99.8% homology). The genomes were sequenced using Illumina HiSeq 2000 paired-end technology at BGI, Hong Kong. The R26^T^ genomic reads (652 Mbp) were *de novo* assembled using DNASTAR NGen v 10.0, which generated 66 contigs, totaling 4.03 Mbp with an average GC content of 35.4%. The Ag1 genomic reads (620 Mbp) were *de novo* assembled in CLC Genomics Workbench v.4.9, which yielded 51 contigs, totaling 4.05 Mbp. The draft genomes were annotated using the NCBI Prokaryotic Genome Automatic Annotation Pipeline (http://www.ncbi.nlm.nih.gov/genome/annotation_prok/), which predicted 3,687 protein coding sequences (CDS) and 44 RNA genes in R26^T^ and 3,648 CDS and 38 RNA genes in Ag1. Strikingly, 112 protein features were identified in the category “Resistance to antibiotics and toxic compounds.” This included drug efflux/transport (36 features); resistance to β-lactam antibiotics, fluoroquinolones, and heavy metals (28, 4 and 25 features, respectively); and 19 additional features involved in resistance to a diverse set of antibiotics. The large genetic capacity against various antibiotics is consistent with the observation that *E. anophelis* has natural antibiotic resistance to several antibiotics (7). Recently, *E. anophelis* was reported as a human pathogen in Central Africa (8) and an outbreak was also seen in an intensive care unit in Singapore (9). In both clinical cases multidrug resistance was reported. Further analysis of the genomic background would improve our understanding of antibiotic resistance mechanisms and their significance in shaping a microbial community in natural environments and the host-associated metagenomic ecosystem (10, 11). Like some *Bacteroides* (12), *E. anophelis* possesses polysaccharide utilization loci (PUL), which suggests the genetic capability to utilize various plant polysaccharides. This implies an intriguing ecological connection with the nectar and plant sap feeding behavior of mosquitoes in nature. The genome of *E. anophelis* plus other bacterial genomes that Xu and collaborators isolated from the mosquito guts, *Pseudomonas* sp. (13) and *Enterobacter* sp. (14), will serve as references for subsequent characterization of the mosquito gut microbiome and its impact on *Anopheles gambiae* life traits. Additionally, the pathogenic and multiresistant nature of the bacteria prompts investigations of the vector potential of mosquitoes for *E. anophelis* transmission to humans.

### Nucleotide sequence accession numbers.

The draft genome sequences of strains R26^T^ and Ag1 are available in DDBJ/EMBL/GenBank under the GenBank accession numbers ANIW00000000 and AHHG00000000, respectively.
